# Nonrapid eye movement sleep and risk for autism spectrum disorder in early development: A topographical electroencephalogram pilot study

**DOI:** 10.1002/brb3.1557

**Published:** 2020-02-09

**Authors:** Jessica Page, Caroline Lustenberger, Flavio Frӧhlich

**Affiliations:** ^1^ Department of Psychiatry University of North Carolina at Chapel Hill Chapel Hill North Carolina; ^2^ Carolina Center for Neurostimulation University of North Carolina at Chapel Hill Chapel Hill North Carolina; ^3^ Department of Communication Sciences and Disorders Northwestern University Evanston Illinois; ^4^ Department of Health Sciences and Technology Institute of Movement Sciences and Sport ETH Zurich Zurich Switzerland; ^5^ Department of Cell Biology and Physiology University of North Carolina at Chapel Hill Chapel Hill North Carolina; ^6^ Neuroscience Center University of North Carolina at Chapel Hill Chapel Hill North Carolina; ^7^ Department of Neurology University of North Carolina at Chapel Hill Chapel Hill North Carolina; ^8^ Department of Biomedical Engineering University of North Carolina at Chapel Hill Chapel Hill North Carolina

**Keywords:** autism spectrum disorder, high‐density electroencephalogram, infants, nonrapid eye movement sleep, sleep spindles, theta, toddlers

## Abstract

**Objective:**

Autism spectrum disorder (ASD) is a pervasive neurodevelopmental disorder that emerges in the beginning years of life (12–48 months). Yet, an early diagnosis of ASD is challenging as it relies on the consistent presence of behavioral symptomatology, and thus, many children are diagnosed later in development, which prevents early interventions that could benefit cognitive and social outcomes. As a result, there is growing interest in detecting early brain markers of ASD, such as in the electroencephalogram (EEG) to elucidate divergence in early development. Here, we examine the EEG of nonrapid eye movement (NREM) sleep in the transition from infancy to toddlerhood, a period of rapid development and pronounced changes in early brain function. NREM features exhibit clear developmental trajectories, are related to social and cognitive development, and may be altered in neurodevelopmental disorders. Yet, spectral features of NREM sleep are poorly understood in infants/toddlers with or at high risk for ASD.

**Methods:**

The present pilot study is the first to examine NREM sleep in 13‐ to 30‐month‐olds with ASD in comparison with age‐matched healthy controls (TD). EEG was recorded during a daytime nap with high‐density array EEG.

**Results:**

We found topographically distinct decreased fast theta oscillations (5–7.25 Hz), decreased fast sigma (15–16 Hz), and increased beta oscillations (20–25 Hz) in ASD compared to TD.

**Conclusion:**

These findings suggest a possible functional role of NREM sleep during this important developmental period and provide support for NREM sleep to be a potential early marker for ASD.

## Graphical abstract

Early diagnosis of autism spectrum disorder (ASD) is challenging as such; there is a growing interest in detecting early brain markers of ASD. Here, we examine the EEG of nonrapid eye movement (NREM) sleep in the transition from infancy to toddlerhood. We found topographically distinct decreased fast theta oscillations (5–7.25 Hz), decreased fast sigma (15–16 Hz), and increased beta oscillations (20–25 Hz) in ASD.

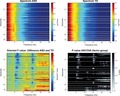

## 1 INTRODUCTION

Autism spectrum disorder (ASD) is a heterogeneous neurodevelopmental disorder with impairments in social communication and restricted/repetitive behaviors DSM‐V (2013), and with increased familial risk upwards to 20% (Ozonoff et al., 2011). Although 1 in 59 children is diagnosed with ASD (Baio et al., 2018) and behavioral symptoms are present in the first 2 years of life (Ozonoff, 2010; Ozonoff et al., 2011), the average age of diagnosis is 4 years (Baio et al., 2018). The diagnosis of ASD in infancy is challenging as it relies on behavioral measures that depend on the child's motivation, professional clinical judgment, and the presence of symptomatology (Liptak et al., 2008) that becomes more pronounced with age (Barton, Dumont‐Mathieu, & Fein, 2012; Filipek et al., 1999; Ventola et al., 2007). Yet, the early diagnosis of ASD (e.g., before the age of 3) is a key factor to enable access to early interventions that could promote improved developmental outcomes (Dawson et al., 2012). As such, identifying neurobiological markers that could aid detection of ASD early in development is of fundamental interest.

Brain oscillations investigated with electroencephalogram (EEG) within nonrapid eye movement (NREM) sleep, such as sleep spindles and slow waves, reflect anatomical and physiological features of brain circuits, and might therefore provide a window to detect alterations in brain development (Buchmann et al., 2010; Ringli & Huber, 2011). These NREM features have been associated not only with anatomical substrates but also with behavioral outcomes such as intelligence (Geiger, Huber, Kurth, & Ringli, 2011), visual perception (Bang, Khalilzadeh, Hämäläinen, Watanabe, & Sasaki, 2014), memory (Chatburn et al., 2013; Fogel, Fogel, Nader, Cote, & Smith, 2007), motor skills (Kurth et al., 2012; Lustenberger et al., 2017), language, social, and cognitive functioning in typical development (Page, Lustenberger, & Frohlich, 2018). We previously established evidence that NREM sleep spectral features undergo developmental changes, in a very early window from 12 to 30 months (Page et al., 2018), when children begin to show signs for developmental concerns. Besides profound changes in the sigma range (10–16 Hz, capturing sleep spindles), theta power also changed across age. Importantly, using high‐density EEG (hdEEG) we have illustrated that these age‐related changes have specific topographic features. In addition, these frequency bands were associated with social and cognitive development at this age (Page et al., 2018). Thus, investigating spectral features of NREM sleep may provide clues to capture divergence in early brain development (Clawson, Durkin, & Aton, 2016; Farmer et al., 2018).

Several studies provide preliminary evidence that features of NREM sleep are altered in neurodevelopmental disorders such as attention deficit hyperactivity disorder (ADHD; Arns & Kenemans, 2014; Ringli et al., 2013), and ASD (Farmer et al., 2018; Limoges, Mottron, Bolduc, & Berthiaume, 2005; Rochette, Soulières, Berthiaume, & Godbout, 2018; Tessier et al., 2015). These alterations suggest atypical development, and identifying these changes in conjunction with other NREM features may indicate transdiagnostic risk of a neurodevelopmental disorder as ASD. Research with adults and children with ASD has found fewer sleep spindles (Farmer et al., 2018; Tessier et al., 2015) and reduced delta activity (Rochette et al., 2018) suggesting anomalies in the thalamocortical network, the anatomical substrate of sleep spindles and delta activity (De Gennaro & Ferrara, 2003; Steriade, 2003). Disruptions in the functional integrity of NREM sleep could be why there are fewer sleep spindles in populations with ASD (Farmer et al., 2018; Tessier et al., 2015) and reduced spindles as well as other altered brain oscillations during NREM sleep may account for observed differences between ASD and typical development. So far, no previous studies have exclusively examined NREM spectral features in infants and toddlers with or at high risk for ASD (referred as an infant sibling of an immediate family member with ASD and thus are at high risk to develop ASD). However, for detection of markers that diverge between ASD and typical development very early on in life, investigations in this time window are crucial.

Thus, the aim here is to build on these findings and characterize the topography of brain network dynamics during NREM sleep using hdEEG during a daytime nap between typically developing infants and toddlers (a subgroup from our previous publication and those who meet ASD criteria [based on “gold standard” instruments]). Topographical and spectral characteristics of NREM sleep may have an underlying role in development, and potential differences may provide insight into the early indicators of ASD.

## 2 METHODS

### 2.1 Participants

Participants were 13‐ to 30‐month‐old infants and toddlers. In total, 20 (13 typically developing [TD] five males, eight females; and seven ASD five males, two females) infants and toddlers (mean age = 21.8, *SD* = 6.3, range = 13–30, in months) participated in this study. The 13 TD participants were part of our previously published study (Page et al., 2018) and selected to match on age (in months) with the participants in the ASD group. We had several TD participants that were similar in age, and to reduce selection bias, we therefore included multiple TD participants that matched on age with a participant in the ASD group. A phone interview with the primary caregiver (parent) was used to ensure eligibility for participation. Inclusion criteria required that participants were described as sleeping between 8 and 14 hr at night, as habitual nappers (nap a minimum 5 days per week), one nap per day, free of known sleep disorders, and free of medication (affecting sleep/daytime alertness/the circadian system) at the time of study participation. Due to considerable developmental change occurring between infancy and toddlerhood, and the presentation of ASD symptomatology in this age range, two different screening tools were used to provide a more sensitive assessment of risk for ASD. Participants 13–16 months were screened with the First Year Inventory (FYI; Turner‐Brown, Baranek, Reznick, Watson, & Crais, 2013), and participants 17–30 months were screened with the M‐CHAT‐R/F (Modified Checklist for Autism in Toddlers, Revised with Follow‐Up; Robins, Fein, & Barton, 2009) to determine risk for ASD. All the participants identified as high risk (infant sibling of an immediate family member with diagnosed ASD) and participants with a formal ASD diagnosis met risk criteria (e.g., scores of 14–25 on the FYI or 8–20 on the M‐CHAT‐R/F) to be part of the ASD group. All of the high‐risk participants (three participants out of the ASD group) scored as having moderate‐to‐severe concern for ASD (scores of 14–20, determined by the Autism Diagnostic Observation Schedule 2nd edition [ADOS‐2; Lord, DiLavore, & Gotham, 2012]) and were grouped with participants with a formal ASD diagnosis (also scoring moderate‐to‐severe) and from here on, referred to as ASD. All TD participants passed the FYI or the M‐CHAT‐R/F (e.g., scores of 0–2) and did not meet criteria for ASD (determined by the ADOS‐2). All participants were born at full gestation. Exclusion criteria were a reported history or presence of epilepsy, neurological/metabolic or developmental disability other than ASD, and severe visual or motor impairments that would impede participation. All experimental procedures were explained to parents before they provided written consent as approved by the Institutional Review Board at the University of North Carolina at Chapel Hill (15‐1829). Parents were compensated with cash for their participation in the study. All participants completed a home visit (behavioral assessment) and a laboratory visit. Participant and maternal demographics are shown in Table 1.

**Table 1:** Child and maternal demographics

Participants (*n* = 20)		
	TD (*n* = 13) *n* (%)	ASD (*n* = 7) *n* (%)
Child gender		
Female	8 (61.5)	2 (28.5)
Male	5 (38.5)	5 (71.5)
Race/Ethnicity		
White	10 (76.9)	4 (57.1)
Black/African American	1 (7.7)	3 (42.9)
Asian/Pacific Islander	0 (0.0)	0 (0.0)
Other/Multiracial	1 (7.7)	0 (0.0)
Hispanic/Latino	1 (7.7)	0 (0.0)
Maternal marital status		
Married	12 (92.3)	4 (57.1)
Never married	0 (0.0)	1 (14.3)
Separated	0 (0.0)	1 (14.3)
Divorced	0 (0.0)	1 (14.3)
Widowed	1 (7.7)	0 (0.0)
Household income		
<$25,000	0 (0.0)	2 (28.5)
$25,00–$49,999	4 (30.7)	1 (14.3)
$50,000–$74,999	3 (23.1)	3 (42.9)
$75,000–$99,999	3 (23.1)	1 (14.3)
>$100,000	3 (23.1)	0 (0.0)
Maternal education		
Associates/Vocational	0 (0.0)	1 (14.3)
4‐year degree	6 (46.2)	2 (28.6)
Graduate/professional	7 (53.8)	4 (57.1)

### 2.2 Assessments: home visit and laboratory visit

All assessments were completed during a home visit and a laboratory visit, as previously described, and administered by a clinical professional who was extensively trained to conduct the ASD screeners and assessment tools (Page et al., 2018). During the home visit, participants completed the Mullen Scales of Early Learning (MSEL; Mullen, 1995), the Vineland Adaptive Behavior Scales—2nd Edition (VABS‐2; Sparrow, Cicchetti, & Balla, 2005), and the toddler module of the ADOS‐2 (Lord et al., 2012). The MSEL is a standardized norm‐referenced tool designed to measure cognitive functioning from birth to 68 months. The VABS‐2 is a standardized norm‐referenced tool established to measure daily functioning from birth to 90 years of age. The ADOS‐2 is a semi‐structured, standardized assessment of communication, social interaction, play, and restricted and repetitive behaviors. The ADOS‐2 is the “gold standard” to assess the possibility and severity of ASD by prompting and eliciting behaviors related to a diagnosis of ASD. All participants (both TD and ASD) completed the toddler module of the ADOS‐2.

Prior to the laboratory visit, all families completed a brief demographic questionnaire and a sleep diary, recording all napping and sleeping for 7 days. Families arrived at the laboratory 30 min before their child's typical naptime for the sleep recording.

### 2.3 EEG: recording, analysis, and sleep scoring

During the laboratory visit, all infants/toddlers were recorded with a 124‐ or 128‐channel hdEEG electrode net (Electrical Geodesic, Inc.) and slept in a bed or a pack‐n‐play. The average nap duration (time in bed/pack‐n‐play) was 78 min (range 43–156 min). There was no significant difference between nap duration between ASD and TD (unpaired *t* test, *p* = .72). Furthermore, it is important to note the possible circadian influence, for example, different naptimes, and homeostatic pressure, for example, time between wake and nap, which may confound group‐specific effects. However, there were no significant differences in the naptime between the two groups (naptime relative to 12 p.m. [in min, mean ± *SD*]; TD: 88.5 ± 50.8, ASD: 54.3 ± 74.5, unpaired *t* test: *p* = .24, effect size Cohen's *d*: 0.54) nor the duration of wakefulness before the nap (duration between wake‐up time in the morning and start of the nap [in min, mean ± *SD*]; TD: 341.2 ± 42.1, ASD: 362.9 ± 60.7, unpaired *t* test: *p* = .36, effect size Cohen's *d*: 0.42).

High‐density electroencephalogram data were collected using Cz as the reference electrode. Data were preprocessed as previously described (Page et al., 2018). In short, data were band‐pass filtered (0.1–200 Hz), digitized at 1,000 Hz, and were resampled offline to 250 Hz and preprocessed using the PREP pipeline (Bigdely‐Shamlo, Mullen, Kothe, Su, & Robbins, 2015). The preprocessing procedure included line‐noise removal and robust average referencing (including bad channel removal), and band‐pass filtering (0.5–40 Hz). Artifact rejection was based upon visual scoring and semiautomatic artifact removal as published previously (Page et al., 2018).

Sleep EEG was visually scored in accordance with the American Academy of Sleep Medicine (AASM; Iber et al., 2007) for sleep stages (20‐s epochs F4A1, C4A1, O2A1) by two expert scorers. Any discrepancies were resolved by mutual agreement. We specifically focused on scoring and analysis on NREM sleep as the majority of the nap contained NREM sleep, on average 52 min (range 22–133 min). Only N2 and N3 were included in the NREM spectral analysis due to missing electrooculography (EOG) and electromyography (EMG), which made it difficult to distinguish between N1 and REM sleep. Moreover, N1 represents a transitionary state between wake and NREM sleep and does not include sleep spindles or slow‐wave features. Spectral analysis was performed as previously described (Page et al., 2018). For all channels, the fast Fourier transform (Hanning window, 20‐s epochs, average of five 4‐s windows) was used. All 20‐s spectral power values were averaged for all artifact‐free NREM episodes and used for further analysis. The number of included artifact‐free NREM episodes did not differ between the groups as revealed by an unpaired *t* test (number of 20‐s epochs, mean ± *SD*: TD: 99.5 ± 54.5, ASD: 117.9 ± 69.1, *p* = .52). The number of identified bad electrodes did not differ between the groups, ASD (mean number ± *SD*): 12.9 ± 9.4, TD: 8.7 ± 12.0, paired *t* test *p* = .44.

### 2.4 Statistical analysis

Statistical analyses were conducted in MATLAB 2016a (MathWorks) and SPSS 24.0.0 (IBM Corp.). The small sample size was the result of limited funding and the difficulty of identifying suitable study participants since children receive an ASD diagnosis on average at age 4 (Baio et al., 2018). All participants meeting criteria for ASD were grouped and had a similar age range to TD children (age in months, TD: mean = 21.4, range = 13–30, ASD: mean = 22.6, range = 13–30, two‐sided unpaired *t* test: *p* = .70). Specifically, for every child in the ASD group, we included at least one TD participant with the same age (in months). We used unpaired two‐sided *t* tests to compare age and nap duration differences between the two groups (ASD and TD). Statistical differences between ASD and TD for the subscales of the MSEL, VABS2, and ADOS‐2 were compared using two‐sided unpaired *t* tests. *p*‐values <.05 were considered significant and <.1 trend level, respectively.

Electroencephalogram data (spectral calculations) from the two groups were compared using electrode‐wise analysis of covariance (ANCOVA) with the group as a factor (ASD and TD) and children's age in months as the covariate (to reduce variability, since we previously observed strong age‐related changes in this age window; Page et al., 2018). EEG data were log‐transformed before statistical analysis to approximate normal distribution. In order to control for multiple comparisons in the topographical plot, we applied a conservative threshold approach as used in Wilhelm et al. (2014). By chance, around 6 electrodes may become significant when 109 electrodes are compared (not including marginal/face electrodes). Thus, we additionally highlighted the clusters that included more than 6 significant electrodes and refer to them as robust significant clusters. This approach is very conservative because the likelihood of these electrodes being all neighbors is much lower, and therefore, the chance of a type II error increases. In order to provide more specific details and exploratory information in this pilot study, we highlight the other significant electrodes and provide the effect size of the ANCOVA by reporting omega squared (*ω* ^2^). Please note that *ω* ^2^ around 0.01 is a small effect size, around 0.06 a medium effect size, and around 0.13 a large effect size.

An exploratory analysis of the relationship between scores on the ADOS‐2 (severity of autism) and robust significantly different EEG spectral power clusters was performed in the ASD group using Pearson correlation.

## 3 RESULTS

### 3.1 Performance on the behavioral assessments

Table 2 shows descriptive statistics examining performance on the MSEL, VABS, and ADOS‐2. As expected, both receptive language and expressive language show significant differences between TD and ASD. In our study sample, all behavioral domains exhibit significant differences in performance, except MSEL visual reception with trend‐level (*p* < .1) differences and the VABS Motor with no significant difference (*p* > .1) between TD and ASD. Given that a defining feature of ASD is delays or impairments in social communication, the significant differences in both expressive language and receptive language (MSEL), and communication and socialization (VABS) between TD and ASD were expected.

**Table 2:** ASD and TD distribution of performance

	TD group (*n* = 13)		ASD group (*n* = 7)		Effect size Cohen's *d*	*p‐*value
	Mean	*SD*	Mean	*SD*		
MSEL	100.7	10.9	73.9	16.4	1.92	<.001**
Visual reception	46.5	6.9	40.1	7.9	0.86	.079†
Fine motor	50.9	9.6	38.7	15.2	0.96	.040*
Receptive language	46.9	9.3	31.4	10.6	1.55	.003**
Expressive language	55.2	11.9	32.9	12.6	1.82	.001**
VABS	94.0	13.6	79.7	10.9	1.16	.028*
Communication	97.2	14.9	79.9	14.8	1.16	.023*
Daily living	93.7	14.6	79.3	12.3	1.07	.040*
Socialization	94.9	9.4	81.4	12.0	1.25	.012*
Motor	95.1	10.0	90.3	9.8	0.48	.318
ADOS‐2	0.7	1.0	17.3	2.6	8.43	<.001**

Abbreviations: ADOS‐2, Autism Diagnostic Observation Schedule—2nd Edition; MSEL, Mullen Scales of Early Learning Composite; VABS, Vineland Adaptive Behavior Scales—Second Edition composite.

^†^ Trend‐level *p*‐value < .10, *significant *p*‐value < .05, **p‐value < .005.

### 3.2 Spectral features in NREM sleep differentiate TD and ASD

**Figure 1:**
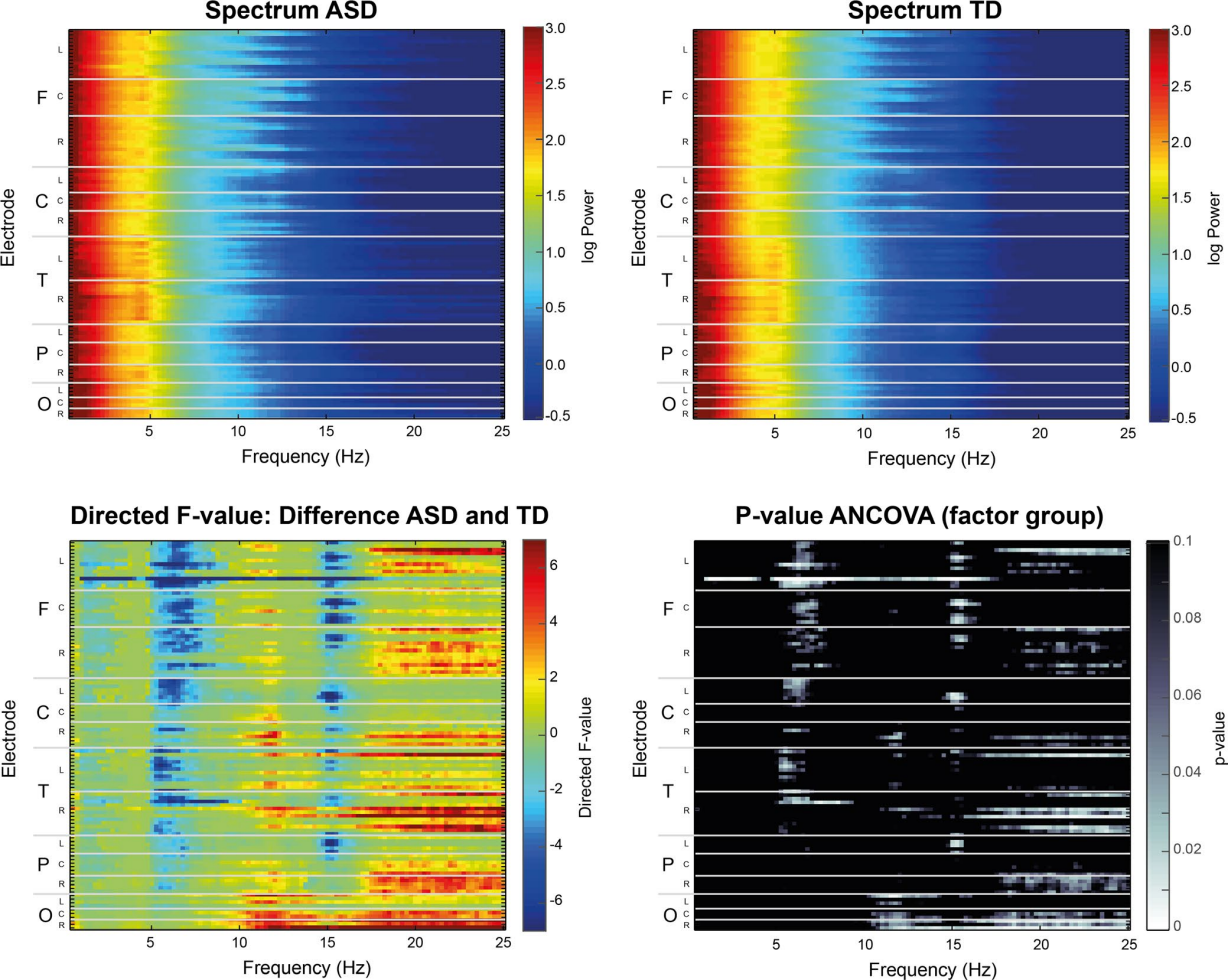
Difference in EEG power in NREM sleep between autism spectrum disorder (ASD) and typically developing (TD) infants and toddlers for all EEG channels (*y*‐axis) and frequency bands between 0.5 and 25 Hz (*x*‐axis). First row depicts spectrogram for both ASD (left) and TD (right) group. Second row illustrates the results of the statistical comparison between ASD and TD. Left: Heat‐map of ANCOVA results for NREM EEG power values between ASD and TD, directed *F*‐value for factor group. Warm colors illustrate power values that are more pronounced for ASD than TD, cold colors vice versa. Right: ANCOVA *p*‐value for factor group. For all plots, the *y*‐axis depicts all EEG electrodes sorted by specific regions (F = frontal, C = central, T = temporal, P = parietal, O = occipital) and hemispheric locations (L = left, C = central, and R = right)

**Figure 2:**
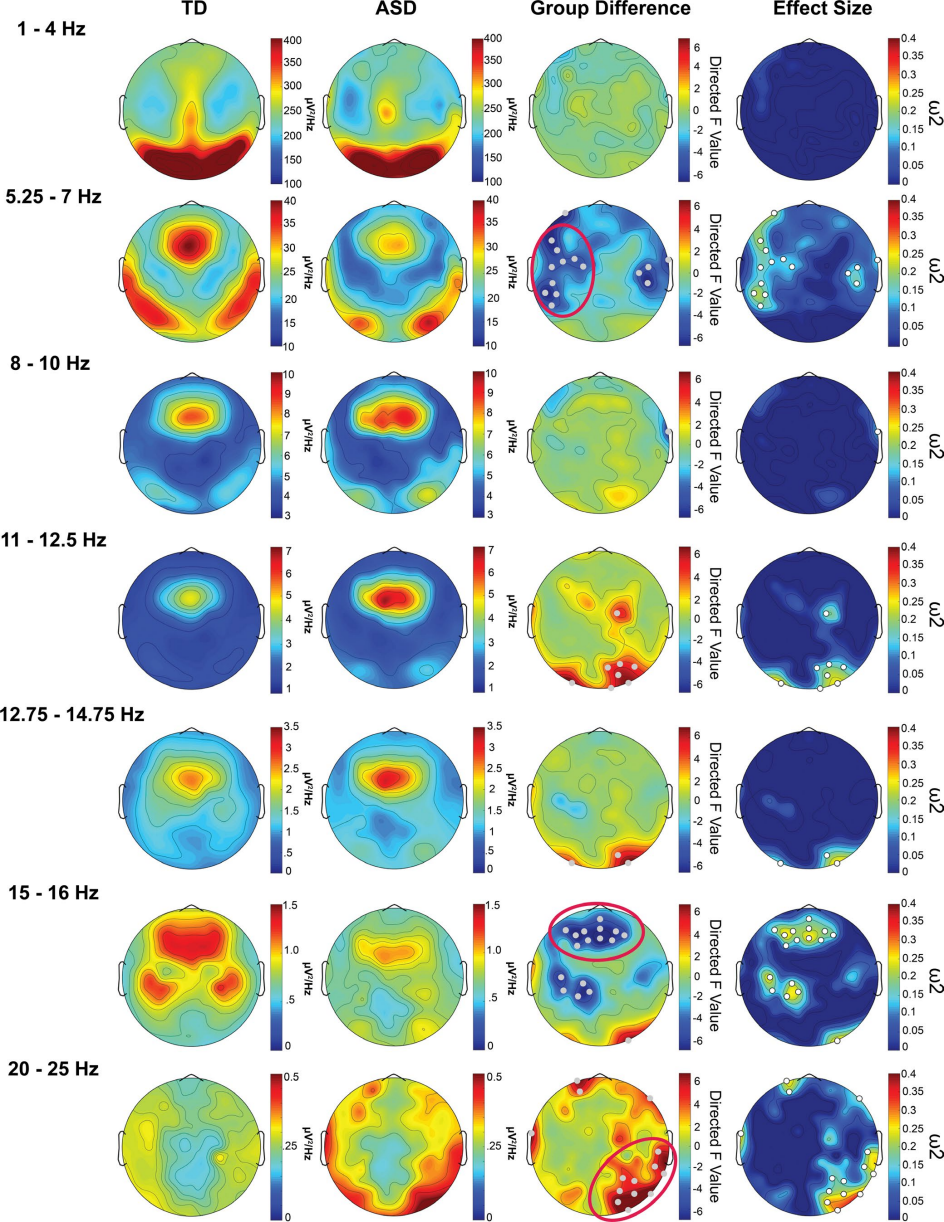
Topographical illustration of spectral power (delta [1–4 Hz], fast theta [5.25–7 Hz], alpha [8–10 Hz], sigma [11–12.5 Hz, 12.75–14.75 Hz, and 15–16 Hz], and beta [20–25 Hz] frequency bands) for each group and their differences during NREM sleep. The first column shows the power values for the typically developing (TD) group and the second column for the autism spectrum disorder (ASD) group. The third column illustrates the directed *F*‐value of factor group (ANCOVA), depicting differences between ASD and TD (warm color: ASD > TD, cold colors: TD > ASD). The fourth column illustrates the effect size (omega squared) of the group difference. Gray/white dots in the third and fourth columns indicate electrodes with statistically significant (*p*‐value < .05) group differences not corrected for multiple comparisons. Circles in the group difference plot highlight robust significant clusters that survived a conservative threshold approach (e.g., at least seven significant electrodes in a cluster; Figure 4 provides further illustration of these clusters)

**Figure 3:**
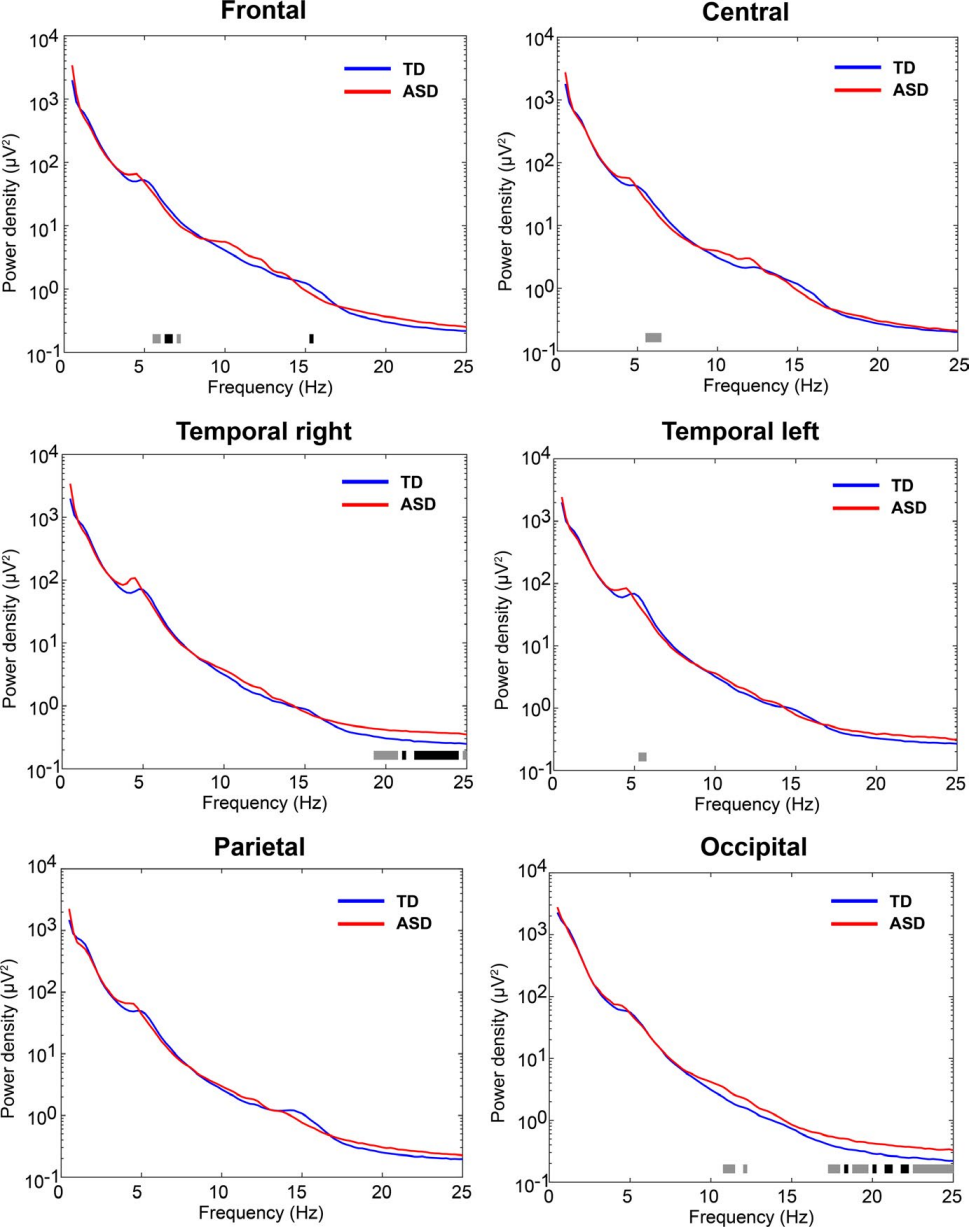
Spectral density for frontal, central, temporal left, temporal right, parietal, and occipital regions across 0.5–25 Hz. Typically developing (TD) and autism spectrum disorder (ASD) are depicted with a blue and red line, respectively. Black bars below the spectral density lines illustrate significant group differences assessed with an ANCOVA (factor group *p* < .05), and trend‐level differences are illustrated with gray bars (factor group .05 ≤ *p* < .1)

**Figure 4:**
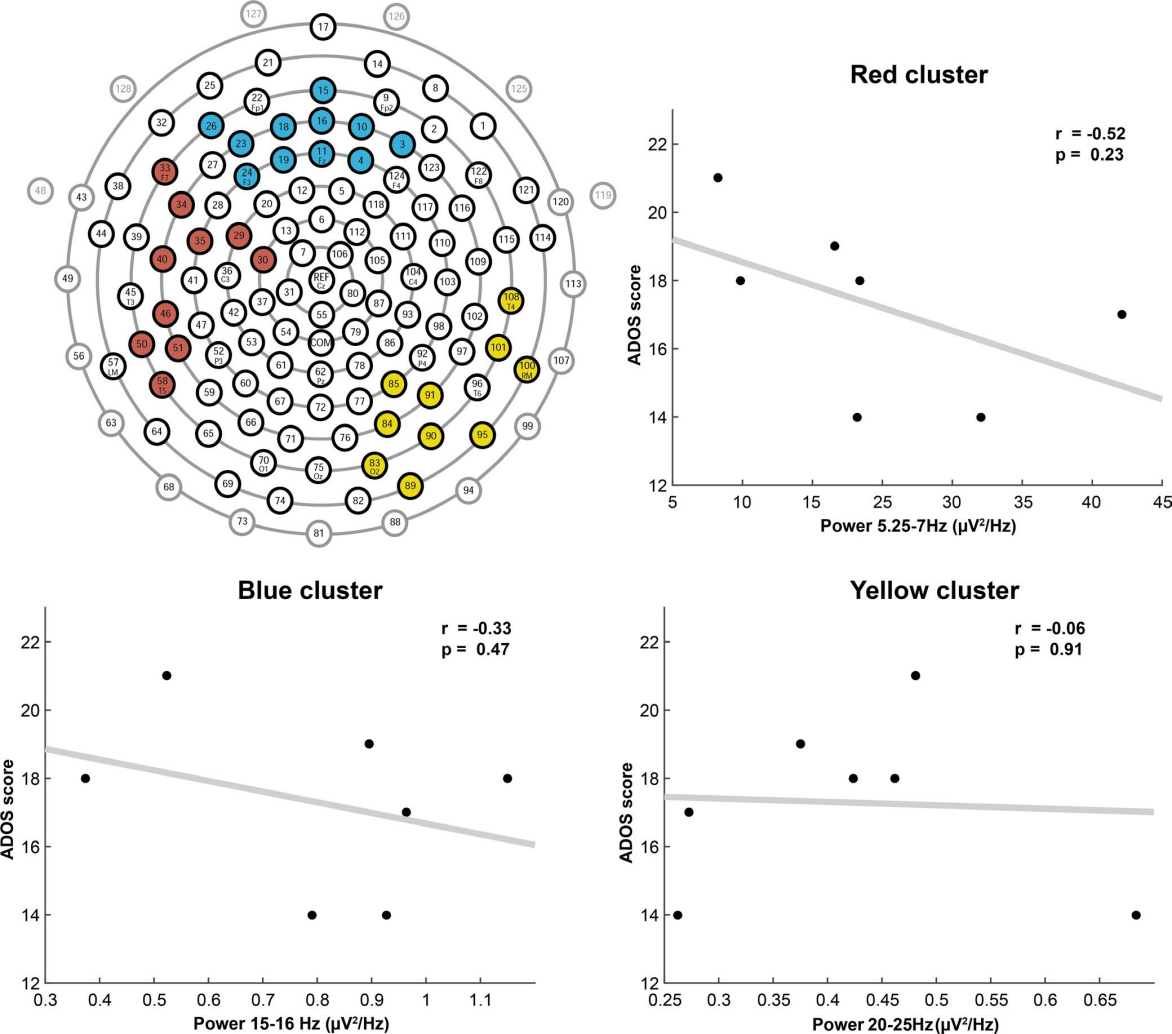
The topographical sensor representation illustrates the robust significant clusters of Figure 2. Gray marked electrodes refer to marginal/face electrodes. Scatterplots show Pearson correlation of the ADOS‐2 scores (autism severity). Each scatterplot illustrates correlations of the ADOS‐2 (*y*‐axis) and spectral power values (*x*‐axis) of the robust significant clusters and respective frequency bands from Figure 2 for all ASD participants. The red cluster refers to the significant electrodes of the 5.25–7 Hz band, the blue cluster denotes the 15–16 Hz band, and the yellow cluster signifies the 20–25 Hz band

The comparison of spectral EEG power during NREM sleep between TD and ASD revealed clear, significant differences for specific frequency bands and regions (Figure 1: Spectra for all electrodes; Figure 2: Topographic representation of power in frequency bands of interest (based on trend‐level/significant differences and bands in‐between); Figure 3: Spectrum by cortical region; and Figure 4: Topographic sensor representation of clusters). Interestingly, activity in the delta range (1–4 Hz, reflecting slow waves) was not different between the groups. However, theta, sigma, and beta range showed significant or trend‐level differences between the groups. The results for these bands are described in detail below.

#### 3.2.1 Theta power

Both groups display a clear theta band during NREM sleep (Figures 1 and 3). However, fast theta power (5.25–7 Hz) was considerably higher in the TD group than the ASD group. Visual inspection shows that both groups have a similar topographical distribution of theta with the most pronounced theta activity over frontal and temporal regions (Figures 1 and 2). Compared to the TD group, there is significantly decreased theta in frontal, central, and some temporal regions in ASD (Figure 1). These regional differences are further shown in Figure 2, with significant electrodes primarily over central and temporal regions. After applying a conservative threshold approach to identify robust significant clusters, only the temporo‐central cluster on the left side remained significant (also see topographical representation in Figure 4, red cluster). Individual power values for these robust clusters are further illustrated in Figure S1. As shown in Figure 3, from visual inspection all examined regions show a faster theta peak in TD and slower theta peak in ASD. Fast theta was statistically significant in frontal regions, with trend‐level differences in frontal, central, and temporal left regions.

#### 3.2.2 Sigma power

The sigma band (capturing sleep spindles) also showed significant differences between TD and ASD. Compared to TD, there was an increase in slower sigma power mainly over occipital and central regions (11–12.5 Hz) and decreased higher frequency sigma power in the ASD group localized over frontal, central, and parietal regions (Figure 1). On a topographical level, the TD group showed increased fast sigma power (15–16 Hz) in frontal and parietal electrode sites (Figure 2). After applying a conservative threshold criterion to identify robust clusters, a cluster over frontal sensor locations survived. Power values for the robust clusters for individual participants are further illustrated in Figure S1. Topographical plots (Figure 2) reveal increased slow sigma power, specifically over occipital electrode sites in ASD. However, after applying the conservative threshold correction, the cluster of electrodes does not remain significant. The increase in slower sigma power and a decrease in faster sigma power in ASD are also present in the spectral density plots (Figure 3). There is significant decreased fast sigma power (for a very narrow frequency band) in frontal regions and trend‐level increased slow sigma power in occipital regions. Compared to TD infants and toddlers, these findings point toward a slower sigma peak in ASD.

#### 3.2.3 Beta power

Another noteworthy difference in the spectral features of NREM sleep was the elevated power in beta frequencies (20–25 Hz) in ASD (Figures 1, 2, 3). Beta power showed significant or trend‐level differences between the groups. These differences are mainly over parietal, occipital, and temporal electrode sites. Both groups have a similar topographical distribution, but significant differences in beta power are localized to a right temporo‐occipital cluster (Figure 2, topographical representation in Figure 4). Visual inspection of the spectral density plots (Figure 3) further shows increased beta activity across all investigated regions, with significantly increased beta in temporal right and occipital regions in ASD.

#### 3.2.4 Spectral power and severity of ASD

To investigate the relationship of NREM spectral power and severity of autism, we performed a correlation analysis between scores on the ADOS‐2 and robust significantly different EEG spectral power clusters in the seven ASD participants (Figure 4). None of the correlations were significant. Yet, the fast theta cluster (5.25–7 Hz) yields a relatively high effect size (*r* = −.52), indicating lower theta power as a potential predictor for increased autism severity. However, the correlations are purely exploratory to demonstrate the type of analyses that can be performed on larger datasets.

## 4 DISCUSSION

This is the first study to characterize NREM sleep with hdEEG in infants/toddlers who meet ASD criteria. The sleep EEG depicts regional and age‐specific changes (Chu, Leahy, Pathmanathan, Kramer, & Cash, 2014) that are associated with neurodevelopmental disorders as ASD (Farmer et al., 2018; Tessier et al., 2015). Among populations with neurodevelopmental disorders, altered features of NREM sleep may be present (Gruber & Wise, 2016), and thus, individuals with ASD may be at increased risk for abnormal NREM characteristics. Our pilot study provides preliminary support for this theory in a very early window of development. Despite the relatively small sample size in our study, statistically clear differences were detected in the NREM spectral features that differentiated TD and ASD. We have previously shown pronounced changes in NREM oscillations occur in the age range of 12–30 months possibly reflecting a sensitive window of pronounced neurodevelopmental change (Page et al., 2018). Therefore, the NREM findings in the ASD group may be capturing a global neurodevelopmental delay reflected in the spectral features. Indeed, several of the frequency bands that showed differences between TD and ASD were also clearly affected by age in our previous study and the spectral features of the ASD group are more closely resembling the younger (<20 months) than older age group (>20 months of age) in our previous study (Page et al., 2018).

In our sample, compared to the TD group, we found a decrease in fast theta power in a cluster of mainly central and temporal electrodes, possibly pointing to a slower theta peak frequency in ASD. Moreover, exploratory correlation analysis showed that theta power in the left centro‐temporal cluster might be a potential predictor for autism severity (indicated by a high effect size, but no significance). Prior research (Chu et al., 2014; Novelli et al., 2016; Sankupellay et al., 2011) shows that during NREM sleep, theta peak frequency and power in higher theta frequency bands are increasing over the first few years of life. Furthermore, we have previously shown in TD infants and toddlers in the same age group that age positively correlated with NREM EEG spectral power in fast theta frequency, showing more pronounced fast theta power with increasing age (Page et al., 2018). Thus, less pronounced fast theta power in ASD may suggest a delay in the development of the circuit that generates activity in this specific frequency band. To the best of our knowledge, no previous research reports differences in theta power during NREM sleep in populations with ASD. Research findings in the wake state in children (5–7 years) with ASD show decreased spectral power and mean coherence in theta in left and right occipital regions during rest and a counting task compared to controls (Kozhushko, 2018; Lushchekina, Podreznaya, Lushchekin, Novototskii‐Vlasov, & Strelets, 2014). Importantly, a clear theta peak during NREM sleep seems to disappear with increasing age (Kurth et al., 2010), and therefore, differences in theta frequencies may not be captured when comparing older age groups (adults). Future research is needed to examine whether the observed changes in theta power and the slowing of the theta peak are specific to NREM sleep and ASD.

Further differences between TD and ASD were observed in the sigma band, the frequency band in which sleep spindles occur. Compared to TD, we identified an increase in sigma power in the slower frequency band of sleep spindles (11–12.5 Hz, occipital cluster, however, not surviving conservative threshold) and a decrease in the faster frequency band of spindles (15–16 Hz, temporo‐occipital cluster) in ASD. Slower spindle frequencies have been found in young children with ASD (Farmer et al., 2018; Tessier et al., 2015). However, more in‐depth analysis and future studies with longer sleep periods are needed to specify how these sigma frequency bands directly relate to individual sleep spindles in infants and toddlers. Interestingly, the spectral and topographic changes between TD and ASD are not clearly pointing to age‐related alterations comparable to the changes we previously observed with age (Page et al., 2018). Altered sleep spindles are widely reported in older populations (e.g., 3–58 years old) with ASD. Compared to TD, participants with ASD (Farmer et al., 2018; Limoges et al., 2005; Tessier et al., 2015) and some research in participants with Asperger syndrome (Godbout, Bergeron, Limoges, Stip, & Mottron, 2000) show decreased spindle characteristics such as spindle density and duration. Furthermore, we found one previous study that differentiated spindle differences in the slow and fast spindle range. Tessier et al. (2015) examined children (6–13 years of age) and also found decreased fast sigma in ASD. More recently, Farmer et al. (2018) reported reduced spindle frequency in young children at ages 2–6 years with ASD, which is supported by our spectral results in the sigma range with increased power in the slower frequency range along with reduced power in the faster frequency range. In our study, we used hdEEG (Chu, 2015; Lustenberger & Huber, 2012), which may be better suited to capture distinct localized differences in the spindle frequency range. Changes in spindle characteristics might point toward physiological and anatomical differences in the thalamocortical system that are involved in the generation of sleep spindles (De Gennaro & Ferrara, 2003; Lüthi, 2014). These electrophysiological differences are congruent with neuroanatomical features associated with ASD (Nair, Treiber, Shukla, Shih, & Müller, 2013). One hypothesis is that the differences in the thalamocortical system between ASD and healthy populations indicate atypical connectivity (Ferradal et al., 2018; Woodward, Giraldo‐Chica, Rogers, & Cascio, 2017) and functioning of thalamocortical neural substrates in ASD (Daoust, Limoges, Bolduc, Mottron, & Godbout, 2004; Rochette et al., 2018), and result in atypical processing of cognitive, attentional, and sensorimotor pathways in children and adults with ASD (Nair et al., 2013). Thalamocortical projections increase over the beginning years of life (Alcauter et al., 2014), and alterations in the thalamocortical connections such as under connectivity to prefrontal, parieto‐occipital, and temporal regions (Nair et al., 2013) suggest atypical connectivity of the developing brain. The current study lends credence to this notion that altered thalamocortical functioning of NREM sleep is also present in infants and toddlers with or at high risk of ASD.

Compared to TD, we found a significant increase in beta activity in a right temporo‐occipital cluster in ASD. In our previous study in TD participants from 12 to 30 months of age, when comparing participants younger than 20 months with participants that are older, younger infants also had higher beta in a similar cluster. In addition, we found a negative correlation of age with beta power in TD (Page et al., 2018). Therefore, the ASD participants in the present study are more closely resembling characteristics of younger TD participants, possibly indicating a developmental delay in ASD. To our knowledge, research has yet to examine altered beta power during NREM sleep in ASD. Interestingly, altered beta activity has been reported in REM sleep (Daoust et al., 2004) and reported for EEG recorded during resting wakefulness in ASD (Kozhushko, 2018; Wang et al., 2013) and Dup15q syndrome (a genetic variation that is associated with ASD; Frohlich et al., 2016). Research with 4‐ to 9‐year‐olds with ASD (Kozhushko, 2018) and research with 2.5‐ to 19‐year‐olds with a copy number of variants of ASD as Dup15q syndrome have shown excessive beta activity during resting state EEG (Frohlich et al., 2016; Kozhushko, 2018). These findings are consistent with the present pilot study where we identified increased beta power in the spectral features of the EEG. It has been hypothesized that excessive higher frequency oscillations such as beta and gamma (Lushchekina et al., 2014; Orekhova et al., 2007) point to imbalances in synaptic excitation and inhibition in the cortex (Orekhova et al., 2007). For example, Orekhova et al. (2007) found increased gamma and beta oscillatory power during wakefulness EEG under sustained visual attention (e.g., bubbles are blown by the experimenter) in ASD. The observed increase in power of higher frequencies of brain activity during wake EEG may also be associated with autism severity (Kozhushko, 2018; Orekhova et al., 2007). This further supports the idea that increased beta activity may be a defining feature of ASD and which is not solely restricted to one state as wake or sleep. Future studies including hdEEG are needed to compare topographical beta activity across different vigilance states in TD and ASD.

Our pilot study further highlights the importance of using EEG with high spatial coverage (hdEEG) to identify regional differences between ASD and TD (Chu, 2015; Lustenberger & Huber, 2012). Our results show that the observed differences in NREM sleep occur in specific regional clusters (Figure 4). Thus, using single‐channel EEG may not capture specific differences between the groups. However, it is important to note that the regions identified on the sensor level might not directly relate to the underlying cortical sources due to distortion by volume conduction.

Only a handful of studies have examined NREM sleep in ASD, and only a couple of studies have examined NREM associations with behavioral measures in young children (Farmer et al., 2018; Tessier et al., 2015). Similar to Farmer et al. (2018), our obtained domain scores and overall standard score (composite) on the MSEL and the VABS differentiated TD and ASD. One of the main differences between groups was the performance on the MSEL. This difference may be due to the group with and at high risk of ASD having possible delays or other comorbidities that have not, yet, been identified. For example, analyses suggest that for some participants with ASD, the low MSEL and VABS quotient could suggest possible comorbidity of intellectual disability. It may be that some of the analyses are capturing characteristics of developmental delay (DD) or disability rather than merely ASD. Due to the vast developmental change that occurs in the age range studied here, variability on cognitive performance in this age span is expected. Thus, future studies are needed to delineate which NREM features are specific to ASD and DD within this age span. This last point is further bolstered by recent research by Farmer et al. (2018) that young children with ASD show reduced sleep spindle features compared to DD and TD. However, there was a strong correlation with spindle density, in which spindle density was greater among children with increased social functioning and higher cognitive quotient. Indeed, altered NREM features such as sleep spindle characteristics are prevalent among neurodevelopmental and psychiatric disorders (Buchmann et al., 2014; Ferrarelli et al., 2007; Gruber & Wise, 2016), and thus, the spectral sleep EEG has transdiagnostic utility in which altered oscillatory features may serve as red flags warranting additional follow‐up. In other words, altered NREM sleep may be a hint that something is of concern, and paired with standardized screening and behavioral assessment, such an investigation may increase the sensitivity and specificity to determine risk for ASD.

### 4.1 Limitations

The presented results here are exploratory in nature, and due to the small sample size, there was no correction or very conservative correction (threshold in the topoplots) for multiple comparisons, which could increase the chance for a type I or II error, respectively. However, since this is the first study looking at spectral differences in such a young age group, we believe it is important to illustrate the complete picture (e.g., Figure 1) and also provide effect sizes. Future studies can build on our observations and hopefully validate our findings in larger samples of infants and toddlers with ASD/risk for ASD.

Sleep disorders, which have been widely associated across the lifespan in populations with ASD (Goldman et al., 2011; Krakowiak, Goodlin‐Jones, Hert‐Picciotto, Croen, & Hansen, 2008), could possibly confound our results. To ensure that we were characterizing NREM sleep in ASD and the observed differences were not due to a comorbid sleep disorder, we specifically included infants/toddlers with no parent‐reported sleep problems or pre‐existing sleep disorders. Another confounding factor that may have influenced group differences is circadian or homeostatic influence. Although the reported effect size is medium, and therefore, a potential influence cannot be completely ruled out, it is still unlikely that homeostatic or circadian influences explain the obtained group differences. For instance, we included infants/toddlers who napped once per day which attenuates the concern of possible effects due to homeostatic pressure. Moreover, homeostatic pressure differences between the groups would be expected in the delta range (marker of sleep pressure, marker of homeostatic process S [Achermann, 2004]); however, this was not observed in our current study.

The present pilot study had a small ASD group. Though all children met criteria for ASD (ADOS‐2), four participants had a formal diagnosis and three were at high risk. Although all participants met diagnostic criteria and were receiving therapies reflective of an ASD diagnosis, not all of the participants had a formal diagnosis. Due to the heterogeneity of the disorder and the onset of symptoms, a larger sample is needed for targeting subgroups allowing for increased precision and identifying distinct populations (Chawarska et al., 2014; Zwaigenbaum et al., 2015). Future research is needed to examine features of NREM sleep in ASD as well as across various categories of DD and other populations with genetic variations of ASD. Including participants with DD or a genetic variant such as Dup15q syndrome (Frohlich et al., 2016) could help decipher if alterations in NREM features are specific to ASD with idiopathic causes or shared secondary features. Such a comparison could increase the sample size of the study and thus increase statistical power, and including children with identified DD or disabilities would allow for further demarcation of subgroups (Walsh, Elsabbagh, Bolton, & Singh, 2011). Furthermore, including infants and toddlers with various risk factors and predispositions for DD would provide valuable insight into the underlying etiologies of ASD as well as other neurodevelopmental disorders.

An additional consideration is that we solely examined NREM sleep. Without EMG and high‐quality EOG data, it is difficult to confidently identify REM. All recordings occurred during a midday nap, and thus, most infants/toddlers had only NREM sleep. To better capture NREM and REM sleep and homeostatic and temporal changes over a sleep period, overnight recordings are needed. Given that this study involved recruitment of very young children, it is important to acknowledge both the family and child's schedule and routines. An additional limitation and consideration for future studies are sex differences. Given that ASD is more prevalent in males, it is not surprising that the group with ASD had more males than females. However, for this small sample size, the chi‐square test did not reveal a significant difference in sex distribution between the two groups.

Finally, our presented results do not directly indicate functional significance. We performed a first explorative analysis of the relationship between the robust electrode clusters that show power differences between the groups and the severity of ASD. Yet, due to the small sample of seven participants in the ASD group, our results may not be representative and will benefit from a larger sample. A possible next step, future research is to include more mechanistic approaches with animal models. For example, mouse and rat models of ASD show features of NREM sleep are also implicated and could possibly be used to study the role of sleep in ASD development. In the Neuroligin‐3 knockout rat model, research by Thomas, Schwartz, Saxe, and Kilduff (2017), points to decreased time spent in NREM sleep and changes in different spectral power values, such as decrease beta, gamma power, and increased delta‐theta power. Research by Liu et al. (2017) shows in a similar animal model (mice) for ASD decreased delta, theta, and alpha in NREM sleep, which mirrors some of our findings. However, direct comparison of these findings with those in human participants is very challenging. From here, research can move from mechanistic and correlational approaches to causal, for example, by actively modulating the affected brain oscillations during sleep via auditory stimulation. Noninvasive brain stimulation techniques such as the application of auditory stimuli have been previously and safely applied in children (6–12 years) with epilepsy to modulate specific sleep oscillations (Fattinger et al., 2019). Epilepsy, as well as other types of seizures and sleep disorders, is common comorbidities in ASD (Mannion, Leader, & Healy, 2013; Veatch et al., 2017), and thus, auditory stimulation provides a safe and intriguing outlet in which interventions can be tailored to selectively modulate specific regions at specific frequencies.

## 5 CONCLUSION

This study contributes to our understanding of the sleep topography of NREM sleep in ASD. Moreover, this study builds upon existing research of NREM sleep showing altered NREM features in ASD and provides preliminary support that NREM sleep oscillations may be used as an early indicator of risk for ASD at a young age. Our study represents a promising first step toward the identification of a potential risk marker of ASD in a very early time window. The findings reported here are applicable to research seeking to understand development in this age range and the neurobiological underpinnings of ASD. Future research with increased sample size and multiple longitudinal recordings across infancy and toddlerhood will further elucidate the role of NREM sleep and provide insight into the potential use of the reported spectral characteristics for the early identification of ASD.

## CONFLICT OF INTEREST

FF is the Founder and Chief Scientific Officer of Pulvinar Neuro. The company played no role in this study.

## Data Availability

The data that support the findings of this study are available from the corresponding author upon reasonable request.
