# Supplementary material for: Nonrapid eye movement sleep and risk for autism spectrum disorder in early development: A topographical electroencephalogram pilot study
Source: Brain Behav. 2020 Feb 9;10(3):e01557. doi: 10.1002/brb3.1557 (PMC7066345; doi:10.1002/brb3.1557)
Supplement: Supplementary file 1 [file BRB3-10-e01557-s001.pdf]

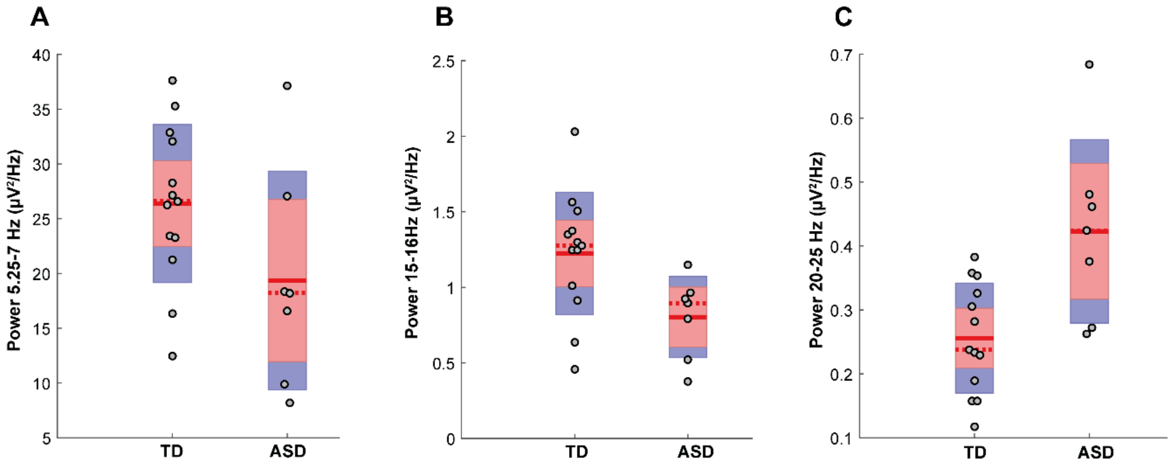

Figure S1: Error bars and individual participant plots for the robust significant clusters marked in Figures 2 and 4. Plots illustrate mean (solid red line), median (red dashes line), 95% confidence interval around the mean (red shaded area) and standard deviation (blue shaded are). Points illustrate the spectral power values for each individual.
